# Ecophysiological responses of native and introduced coastal tree species parasitized by *Cassytha filiformis* in Brunei

**DOI:** 10.1002/pei3.70000

**Published:** 2024-07-22

**Authors:** Roshanizah Rosli, Kushan U. Tennakoon, Faizah Metali

**Affiliations:** ^1^ Environmental and Life Sciences Programme, Faculty of Science Universiti Brunei Darussalam Gadong Brunei Darussalam; ^2^ Institute for Biodiversity and Environmental Research Universiti Brunei Darussalam Gadong Brunei Darussalam; ^3^ Institute of Innovation, Science and Sustainability, Future Regions Research Center Federation University, Berwick Campus Berwick Melbourne Australia

**Keywords:** *Cassytha*, *F*_v_ */F*_m_, gas exchange, net photosynthetic rate, parasitic plants, stomatal conductance, transpiration rate, water‐use efficiency

## Abstract

Hemiparasitic *Cassytha filiformis* commonly infects native host (*Dillenia suffruticosa* and *Melastoma malabathricum*) and introduced host (*Acacia auriculiformis* and *Acacia mangium*) species in threatened heath forests in Brunei. This study aims to investigate the impact of parasitism on the ecophysiology of these host species. This study addresses the research gap in understanding the ecophysiology of *C. filiformis*–host associations, particularly when native and introduced hosts were infected. We generated CO_2_ and light response curves to examine the effects of increasing CO_2_ and light levels of infected and uninfected hosts and examined gaseous exchange, mineral nutrients, and secondary bioactive compounds of host–parasite associations. Infected hosts were negatively impacted by *C. filiformis* as exhibited in the CO_2_ and light response curves, with *C. filiformis*–native host association performing better than introduced species. There were no significant differences in photosynthetic parameters between infected and uninfected hosts, except in *D. suffruticosa*. Certain nutrient contents showed significant differences, but total N, Ca, and K in uninfected hosts were similar to infected hosts. Total phenols and tannins were significantly higher in introduced hosts than native hosts. Our findings asserted that this hemiparasitic vine relies on both its photosynthetic efficiency and nutrient acquisition from its hosts. The parasitism did not significantly hinder the ecophysiological performance of infected hosts, suggesting a plausible co‐existence between the hosts and *C. filiformis*. This study provides essential ecophysiological information for future research on how *C. filiformis* can establish itself without negatively impacting the co‐habitating native hosts.

## Graphical abstract

Our study aims to uncover the impacts of *Cassytha filiformis* on two native and two introduced hosts in terms of photosynthetic parameters, nutrient levels, and secondary bioactive compounds. Significant results were shown in certain nutrient contents, as well as total phenols and tannins in *C. filiformis*–host dynamics. We deduced that parasitism did not significantly hinder the ecophysiological performance of infected hosts, suggesting a plausible co‐existence between the hosts and *C. filiformis*.

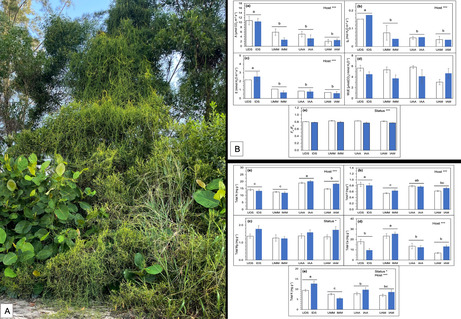

## 1 INTRODUCTION

Parasitic plants play a crucial role in their natural ecosystems and are considered keystone species within their communities (Press & Phoenix, 2005). While they are known to have detrimental effects on host productivity and competitiveness (Fisher et al., 2013), these specialized plants can also be pursued as biocontrol agents, particularly in curbing the spread of invasive and introduced species (Li et al., 2012). Hemiparasites are chlorophyllous parasitic plants that obtain water and mineral nutrition from their hosts while also being able to photosynthesize to a certain extent (Musselman & Press, 1995; Teixeira‐Costa & Davis, 2021). Being both parasitic and autotrophic in nature, hemiparasitic plants are highly responsive to environmental changes and may even become competitive (Těšitel, 2016). The fitness and survival of hemiparasites are particularly dependent on their photosynthetic efficiency and the acquisition of nutrient resources from the hosts' vasculature.

Nutrient transfer in the hemiparasite–host associations has been extensively explored. Studies have reported that mistletoe‐infected hosts have decreased mineral content such as nitrogen (N) compared to uninfected hosts (Muche et al., 2022), leading to eventual mortality as host biomass decreases. Irving et al. (2019) suggested that the hemiparasitic *Phtheirospermum japonicum* preferred carbon (C) over N. As a hemiparasite, *Cassytha* assimilates nutrients from hosts via xylem–xylem connections (Li & Yao, 1992). Cirocco et al. (2018) reported that N and K contents in the alien invasive *Ulex europaeus* were reduced when parasitized by *Cassytha pubescens*.

The reduced photosynthetic capacity in *Cassytha*‐parasitized hosts often leads to a decrease in host biomass (Zhang, Florentine et al., 2022). Numerous physiological studies by Prider et al. (2009, 2011), Shen et al. (2010), and Cirocco et al. (2016a, 2017, 2018) have concluded that the native hemiparasitic vine *C. pubescens* decreased the photosynthetic activities of invasive shrubs, *U. europaeus* and *Cytisus scoparius* in South Australia. Detailed glasshouse investigations have revealed significant negative impacts of the parasite on photosynthetic rates, stomatal conductance, transpiration rate, light‐saturated electron transport rates, and pre‐dawn and midday quantum yields. Consequently, *C. pubescens* was considered a viable biocontrol species for invasive plants. In terms of varying light levels, specifically photosynthetically active radiation, PAR, it has been reported that the infected native *Leptospermum myrsinoides* maintained its photoprotective capacity despite a decrease in the host's foliar pigment concentration (Cirocco et al., 2015). While many studies have investigated the photosynthetic effects of *C. pubescens* on hosts, Prider et al. (2009) reported that the hemiparasitic vine had higher photosynthetic rates, growth rates, and biomass when infecting introduced hosts compared to native host species in South Australia. The photosynthetic characteristics and chlorophyll content of *Cassytha filiformis* have been studied in South Africa by de la Harpe et al. (1979, 1980, 1981), but its physiological interaction with hosts, however, has not been documented.

Elevated CO_2_ levels have been shown to reduce the deleterious effects of parasites on host growth (Dale & Press, 1998; Watling & Press, 2000). Experiments on the influence of increased CO_2_ on the hemiparasitic mistletoe, *Dendrophthoe curvata*, and its host species *Andira inermis*, *Mangifera indica*, and *Vitex pinnata* have also demonstrated similar results with Le et al. (2016) attributing this to the partial dependence of the parasite on host‐derived carbon. However, no studies have been reported on the effects of elevated CO_2_ on *Cassytha*–host associations especially under natural conditions due to the impacts of climate change.

*Cassytha filiformis*, commonly known as the laurel dodder, is the only pantropical species in the *Cassytha* genus belonging to the sub‐family Cassythoideae in Lauraceae and the magnoliid clade (Awang et al., 2018; Weber, 1981; Zhang, Florentine et al., 2022). In Brunei Darussalam, *C. filiformis* is commonly found along the coastal highways where secondary heath (*Kerangas*) forests are located (Rosli, 2014; Tennakoon et al., 2016). In general, heath forests are characterized by their acidic, nutrient‐poor sandy soils and are home to *Kerangas* specialists, such as tropical conifers *Agathis borneensis*, *Gymnostoma nobile*, and *Calophyllum ferrugineum* (Ikbal et al., 2023; Sellan et al., 2022; Wong et al., 2015; Zoletto & Cicuzza, 2022). However, when disturbed, these forests are often dominated by native pioneer species, such as *Rhodomyrtus tomentosa*, *Timonius flavescens*, and *Commersonia bartramia*, and the exotic and highly invasive *Acacia* species, which pose a significant threat to the forest ecosystems (Din et al., 2015; Newbery, 1991; Tuah et al., 2020). This specialized forest type is also threatened by anthropogenic activities such as urban developments and fire disturbances (Din et al., 2015; Jambul et al., 2020; Tuah et al., 2020).

There is an apparent research gap in understanding the physiological interactions in *C. filiformis*–host associations in Southeast Asia. Limited knowledge exists regarding how this pantropical *Cassytha* species responds to environmental changes in terms of gaseous exchange and mineral contents, as well as the consequences of its parasitism on hosts. While many studies examining the interactions between *Cassytha* and its hosts were conducted in greenhouse or controlled laboratory settings, there is limited information available on field studies under natural conditions, specifically focused on the effects of *Cassytha* on host plants. Understanding the ecophysiological responses of host species in the *C. filiformis*–host associations can provide valuable evidence regarding the feasibility of *C. filiformis* as a biocontrol agent against invasive exotic species. Furthermore, it can shed light on the potential implications of this parasitic plant on environmental changes, such as elevated CO_2_ levels associated with climate change. By predicting the responses of *C. filiformis* and its host species to these environmental factors, especially in a threatened habitat, we can gain insights into the broader impacts and ecological dynamics that may arise.

This paper aims to address this gap by presenting the first ecophysiological study on the influence of *C. filiformis* on both heath (*Kerangas*) native and introduced hosts species, with the aim to elucidate the effects of parasitism on the hosts' photosynthetic activities under varying light levels and elevated CO_2_ concentrations, instantaneous gas exchange, chlorophyll fluorescence, mineral nutrients, and secondary bioactive compounds. Concomitantly, this study also examines these parameters in *C. filiformis* when parasitizing various native and introduced hosts. Therefore, this study addressed the following questions: (i) Are there significant differences in the studied parameters between *Cassytha*‐infected and uninfected host species? (ii) Are there significant differences in the studied parameters between native and introduced host species? (iii) Do variations in the performance of *C. filiformis* on different hosts depend on whether the hosts are native or introduced?

## 2 MATERIALS AND METHODS

### 2.1 Study site and species

The study samples were collected from the secondary tropical heath (*Kerangas*) forests located along the coast of Brunei Darussalam, specifically from 4°57′59.99° N, 114°52′33.531° E to 4°59′6.22° N, 114°54′1.472° E, in Northwestern Borneo. Bornean heath forests are found predominantly on podzolized, highly acidic, sandy soils with relatively low macronutrients (Ghazoul & Sheil, 2010; Ibrahim et al., 2022; Jaafar et al., 2016). Details of the study site are in Rosli et al. (2024). The collection period was from December 2021 to June 2022, covering both the relatively wet (December 2021–March 2022) and dry periods (April–June 2022). Brunei has a tropical equatorial climate, with an average monthly temperature of 27.5°C and a total annual rainfall of 3733.2 mm (Brunei Darussalam Meteorological Department, unpublished data).

Experiments were conducted using infected and uninfected hosts: the native *Dillenia suffruticosa* (Dilleniaceae), *and Melastoma malabathricum* (Melastomataceae), as well as the introduced species *Acacia auriculiformis* and *Acacia mangium* (Fabaceae). The study also utilized the vine, *C. filiformis* (hereafter also referred to as *Cassytha*) parasitizing on the respective infected hosts: *D. suffruticosa*, CDS; *M. malabathricum*, CMM; *A. auriculiformis*, CAA; and *A. mangium*, CAM.

**FIGURE 1:**
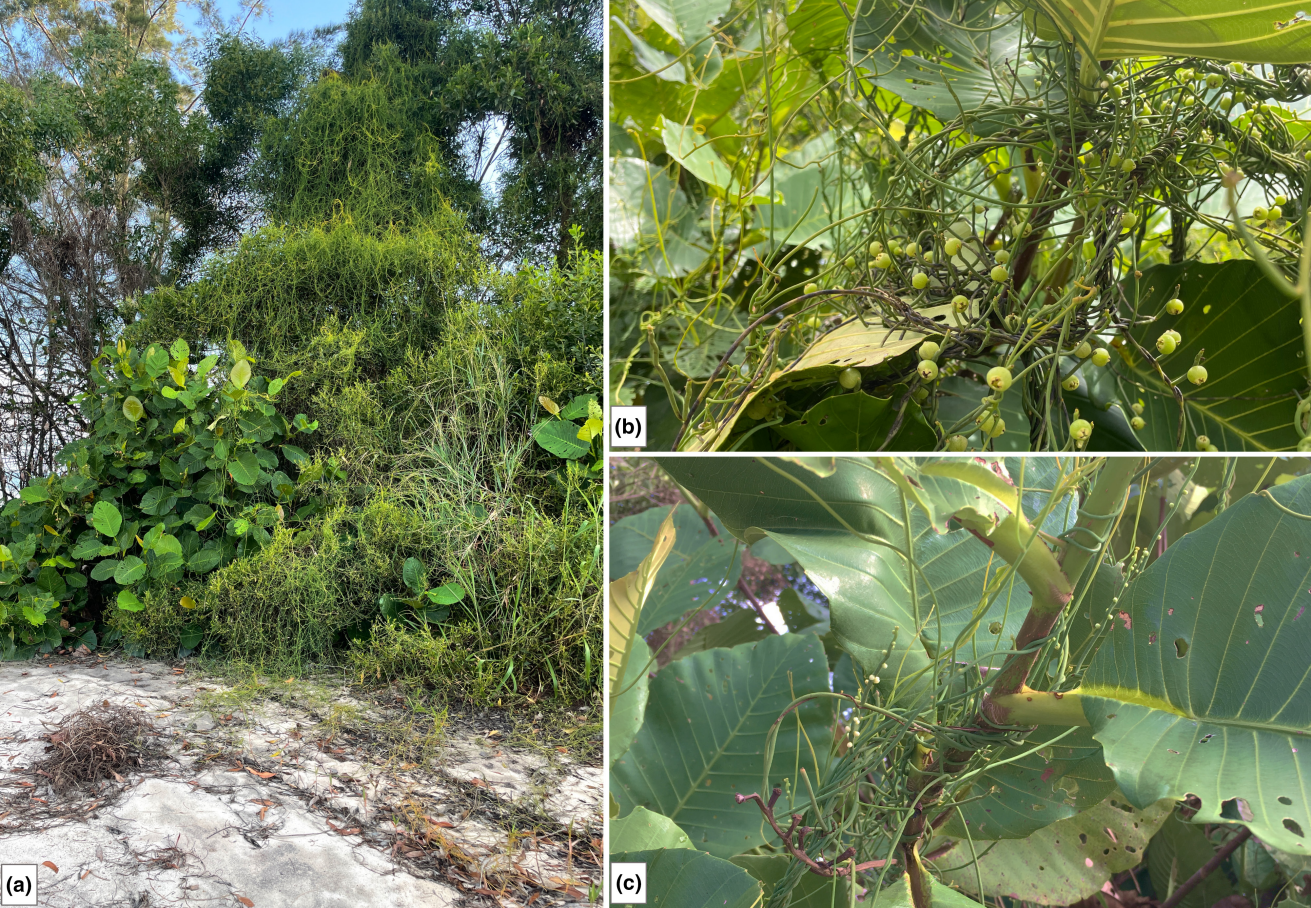
*Cassytha filiformis* heavily infecting several host species at one of the coastal secondary heath forests (a), high degree of fruiting *C. filiformis* on a common secondary native host, *Dillenia suffruticosa* (b), numerous strands of *C. filiformis* vines twining on the stem of *D. suffruticosa* (c).

According to the field surveys on the host range in Brunei, these four species were the most common host species in the study site (Figure 1) (Rosli et al., 2024). *Dillenia suffruticosa* (Griff. ex Hook. f. and Thomson) Martelli. and *M. malabathricum* L. are important native pioneer shrubs that have significant impacts on the secondary succession of tropical forests (Rosli et al., 2024). They are also well‐known for their vast medicinal properties (Armania et al., 2013; Goh et al., 2017). *Acacia auriculiformis* A. Cunn. ex Benth and *A. mangium* Willd. are two most dominant, fast‐growing leguminous tree species native to Australia that were introduced to Brunei in the late 1980s for soil erosion and as a timber plantation tree species, but later became highly invasive, particularly along the coastal highway (Ismail & Metali, 2014; Jambul et al., 2020; Osunkoya & Damit, 2005; Tuah et al., 2020). All four study host species have been reported to co‐exist in the secondary heath forest (Ibrahim et al., 2022).

### 2.2 Gas exchange measurements

Detached, mature, fully expanded, and sun‐exposed leaves or phyllodes were utilized to measure the photosynthesis of parasitized and non‐parasitized hosts, namely, *D. suffruticosa*, *M. malabathricum*, *A. auriculiformis*, and *A. mangium*, following the protocol described by Tang and Wang (2011). All hosts used in this experiment were at a height of more than 0.5 m and parasitized with *C. filiformis* at moderate to high infection levels. Such infection levels entail parasite cover of more than 50% of the host species, according to Rosli et al. (2024). Measurements were conducted ex situ under controlled conditions with 50%–60% relative humidity using a portable gas exchange system (LI‐6400XT; LI‐COR Lincoln, NE, USA). The flow rate in the leaf chamber was maintained at a constant rate of 500 μmol s^−1^, while the leaf temperature was controlled at 25°C. Net photosynthetic rate (*A*), intercellular CO_2_ concentration (*C*_i_), and photosynthetic photon flux density (PPFD) were recorded for the following experiments. Irradiance was provided by a red‐blue light‐emitting diode (LED) source (model 6400‐02B, LI‐COR Lincoln, NE, USA) on a 2 cm × 3 cm chamber (LI‐COR Lincoln, NE, USA) at 1500 μmol photons m^−2^ s^−1^. Chamber CO_2_ concentration (*C*_a_) was maintained at 400 μmol mol^−1^ with a CO_2_ mixer. During each measurement, one leaf per plant and six randomly selected host plants per treatment were sampled as described by Shen et al. (2007, 2010) and Le et al. (2016, 2018).

Two sets of photosynthetic measurements were made. In the first set of measurements, response curves of net photosynthetic rate (*A*) to intercellular CO_2_ concentrations (*C*_i_) were generated by varying the air CO_2_ concentrations (*C*_a_) in the chamber using a CO_2_ mixer at the following values: 50, 100, 150, 250, 350, 400, 450, 500, 700, 950, 1250 μmol mol^−1^. The PPFD on the LED chamber was kept at 1500 μmol photons m^−2^ s^−1^. On the other hand, light response curves were recorded by measuring photosynthesis at PPFD values of 0, 10, 20, 40, 60, 120, 250, 500, 1000, 1500, and 1800 μmol photons m^−2^ s^−1^ and set up as backward curves with at least 120 s for the leaves to acclimate to each set of conditions. A CO_2_ mixer controlled the chamber CO_2_ concentration (*C*_a_) at 400 μmol mol^−1^ throughout the experiment. In the second set of measurements, net photosynthetic rate (*A*) (μmol CO^2^ m^−2^ s^−1^), stomatal conductance (*g*_s_) (mol H_2_O m^−2^ s^−1^), and transpiration rates (*E*) (mmol H_2_O m^−2^ s^−1^) were obtained directly from the portable gas exchange system. Water‐use efficiency (WUE) (μmol CO_2_ mmol H_2_O^−1^) was calculated by *A*/*E* ratio, following the approach described by Farquhar and Richards (1984).

For gaseous exchange measurements in *Cassytha*, green sections of the stem were selected below the young, soft growing “shoot” tip, as described by Prider et al. (2009). The measured area was determined by calculating the surface area of the stem portion clamped inside the opaque conifer chamber (model 6400‐22, LI‐COR Lincoln, NE, USA) exposed to the LED source. A standardized stem area was used for *Cassytha* since the stem diameters were relatively similar. The *Cassytha* stems used in this experiment were sun‐exposed, healthy, and actively parasitizing their respective hosts, which were also used for photosynthesis measurements above.

### 2.3 Chlorophyll fluorescence measurements

Chlorophyll fluorescence was induced by a red light at 3500 μmol m^−2^ s^−1^ with a saturation width of 1.0 s (Strasser et al., 2000) and subsequently measured using a portable chlorophyll fluorometer (OS‐30P+; Opti‐science, Inc., Hudson, NH, USA). Similar to the photosynthetic experiments, detached leaves or phyllodes and their infecting *Cassytha* stems were used and dark‐adapted for 1 h. Chl *a* fluorescence was recorded immediately after treatments, following the methods described by Yu and Ong (2002) and Le et al. (2016). All plant samples were exposed to full sun prior to sampling for dark adaptation (Le et al., 2018; Maxwell & Johnson, 2000).

### 2.4 Mineral and phenolic compounds analysis

Leaves or phyllodes of six individuals of infected and uninfected hosts for all four *Cassytha*–host associations were sampled following Tennakoon et al. (2011, 2014). Parasitizing *Cassytha* stems on the sampled hosts were also collected for nutrient analysis sampling. Young to matured stems were selected for this experiment. Air‐dried and ground plant samples were used to determine the concentrations of total nitrogen (N) and total phosphorous (P). The samples were digested using 98% H_2_SO_4_ and Kjeldahl tablets in a block digester, and the total N and P concentrations were measured using Flow Injection Analyzer (FIA; Model FIAstar 5000, Hoganas, Sweden). For the analysis of total K, Ca, and Mg concentrations, air‐dried and ground samples were acid‐digested with 70% H_2_SO_4_ and H_2_O_2_, following the adapted methods from Allen et al. (1989) and Metali et al. (2015) and measured using a Flame Atomic Absorption Spectrophotometer (AAS; Thermo Scientific iCE 3300, Sydney, Australia).

Total phenols and tannins were measured for both infected and uninfected hosts and their parasitizing *Cassytha* samples for all four *Cassytha*–host associations. Air‐dried and ground plant samples were used for both antioxidant analyses. Samples were extracted using 95% methanol and analyzed using modified methods by Ainsworth and Gillespie (2007). Total soluble phenolics in the extracts were determined with Folin–Ciocalteu reagent using gallic acid as the standard phenolic compound. The absorbance was read at 750 nm wavelength. The presence of tannins in the samples was determined following the modified procedure described by Toth and Pavia (2001) and Makkar et al. (2007).

### 2.5 Statistical analysis

The statistical analyses were performed using the R statistical program version 4.2.2 (R Core Team, 2022). Photosynthetic parameters (*A*, *g*_s_, *E*, WUE), chlorophyll *a* fluorescence (*F*_v_/*F*_m_ ratio), nutrient analyses (total N, P, Mg, Ca, and K), and total phenols and tannins were statistically tested for normality and homogeneity of variance using Shapiro–Wilk and Levene tests, respectively. In cases where these assumptions were violated, the data were subjected to log_10_ transformations. Two‐way analysis of variance (ANOVA) and post hoc Tukey's HSD tests were used to determine: (1) the effects of host species (*D. suffruticosa*, *M. malabathricum*, *A. auriculiformis*, and *A. mangium*) represented as “Host” and infection status (infected and uninfected) represented as “Status” on photosynthetic parameters (*A*, *g*_s_, *E*, WUE), chlorophyll *a* fluorescence (*F*_v_/*F*_m_ ratio), nutrient analyses (total N, P, Mg, Ca, and K), and total phenols and tannins; (2) the effects of *C. filiformis* parasitizing the respective studied hosts (*D. suffruticosa* or CDS, *M. malabathricum* or CMM, *A. auriculiformis* or CAA, and *A. mangium* or CAM) represented as “*Cassytha*” with the infected host's origin categorized as either native for CDS and CMM or introduced for CAA and CAM represented as “Origin” on the same set of parameters. All analyses were conducted at 5% significance level. All mean values related to photosynthetic parameters (*A*, *g*_s_, *E*, WUE), chlorophyll *a* fluorescence (*F*_v_/*F*_m_ ratio), nutrient analyses (total N, P, Mg, Ca, and K), and total phenols and tannins are presented in Tables S1–S4. All abbreviations used for experimental materials (samples) and measurement variables are listed in Table 1.

**TABLE 1:** List of abbreviations used for experimental materials (samples) and measurement variables.

	Abbreviation	Name
Samples	DS (IDS, UDS)	*Dillenia suffruticosa* (Infected, uninfected)
	CDS	*Cassytha filiformis* parasitizing on *Dillenia suffruticosa*
	MM (IMM, UMM)	*Melastoma malabathricum* (Infected, uninfected)
	CMM	*Cassytha filiformis* parasitizing on *Melastoma malabathricum*
	AA (IAA, UAA)	*Acacia auriculiformis* (Infected, uninfected)
	CAA	*Cassytha filiformis* parasitizing on *Acacia auriculiformis*
	AM (IAM, UAM)	*Acacia mangium* (Infected, uninfected)
	CAM	*Cassytha filiformis* parasitizing on *Acacia mangium*
Measurements	*A*	Net photosynthetic rate
	*C*_i_	Intercellular CO_2_ concentration
	*C*_a_	Chamber air CO_2_ concentration
	PPFD	Photosynthetic photon flux density
	*g*_s_	Stomatal conductance
	*E*	Transpiration rate (*E*)
	WUE	Water‐use efficiency
	N	Total nitrogen concentration
	P	Total phosphorus concentration
	K	Total potassium concentration
	Ca	Total calcium concentration
	Mg	Total magnesium concentration

## 3 RESULTS

### 3.1 Photosynthetic CO_2_ and light response curves of host and parasitic plants

**FIGURE 2:**
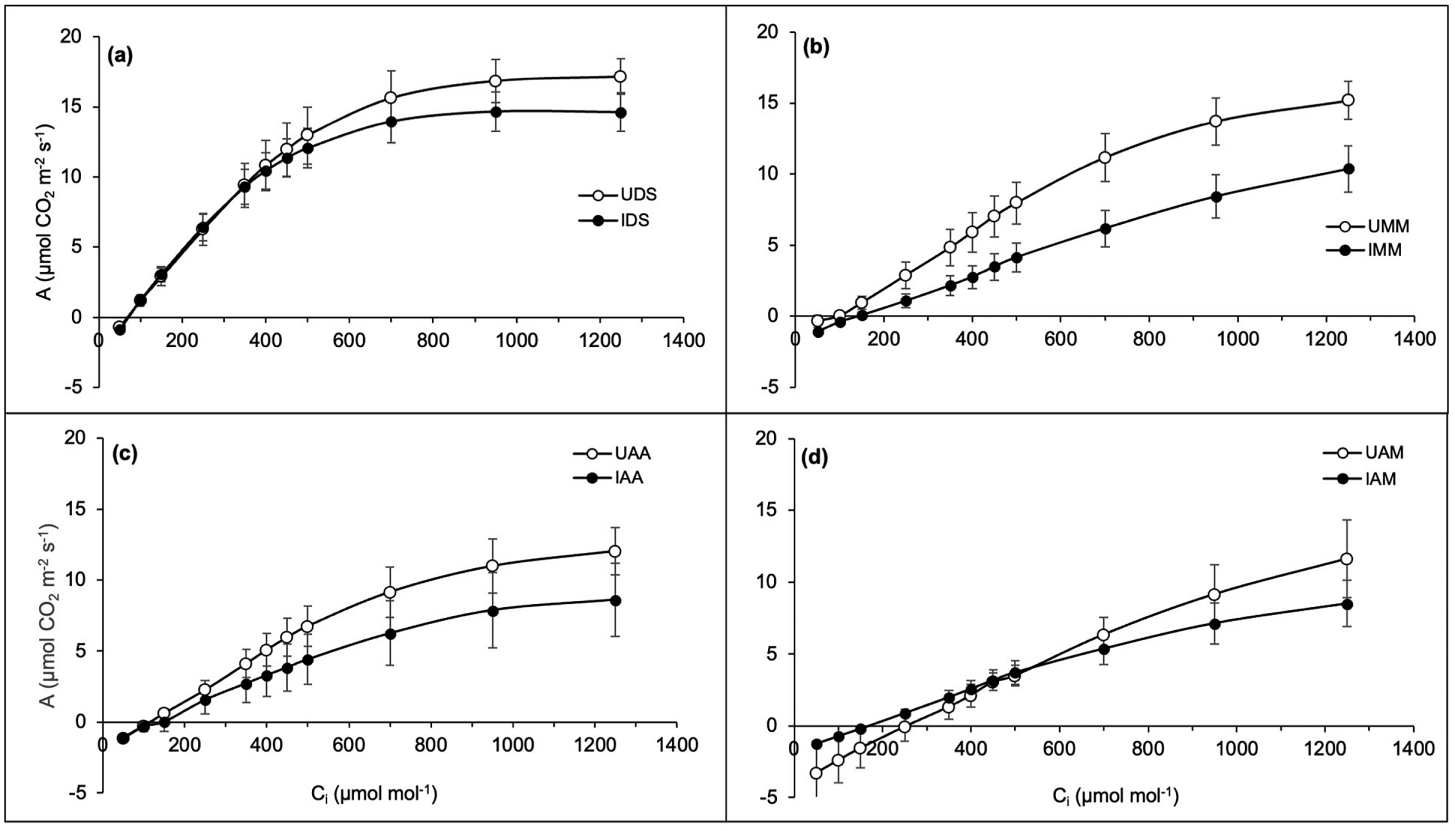
Photosynthetic CO_2_ response (*A*–*C*_i_) curves of uninfected (open circle) and infected hosts (black circle) infected by *Cassytha filiformis*: (a) *Dillenia suffruticosa* (DS), (b) *Melastoma malabathricum* (MM), (c) *Acacia auriculiformis* (AA), (d) *Acacia mangium* (AM). The measurements were taken at PPFD of 1500 μmol photons m^−2^ s^−1^ and leaf temperature of 25°C. The data are expressed as mean values ± standard error, SE with *n* = 6, except for UMM (*n* = 5) and UAM (*n* = 4).

The photosynthetic CO_2_ response curves (*A*–*C*_i_) for all studied hosts (i.e., native *D. suffruticosa* and *M. malabathricum*, and the exotic *A. auriculiformis* and *A. mangium*) exhibited similar patterns with parasitized hosts had lower photosynthetic performance, *A*, than their respective uninfected hosts (Figure 2a–d). In *D. suffruticosa*, the curve gradually decreased when the infected samples were negatively affected at *C*_i_ of about 400 μmol mol^−1^ but reached saturation point after 950 μmol mol^−1^ (Figure 2a). Phyllodes of infected *A. mangium* showed a slightly different pattern, in which they had higher *A* than uninfected hosts when *C*_i_ <500 μmol mol^−1^ (Figure 2d). As the *C*_i_ increased, the uninfected *A. mangium* outperformed the parasitized hosts.

**FIGURE 3:**
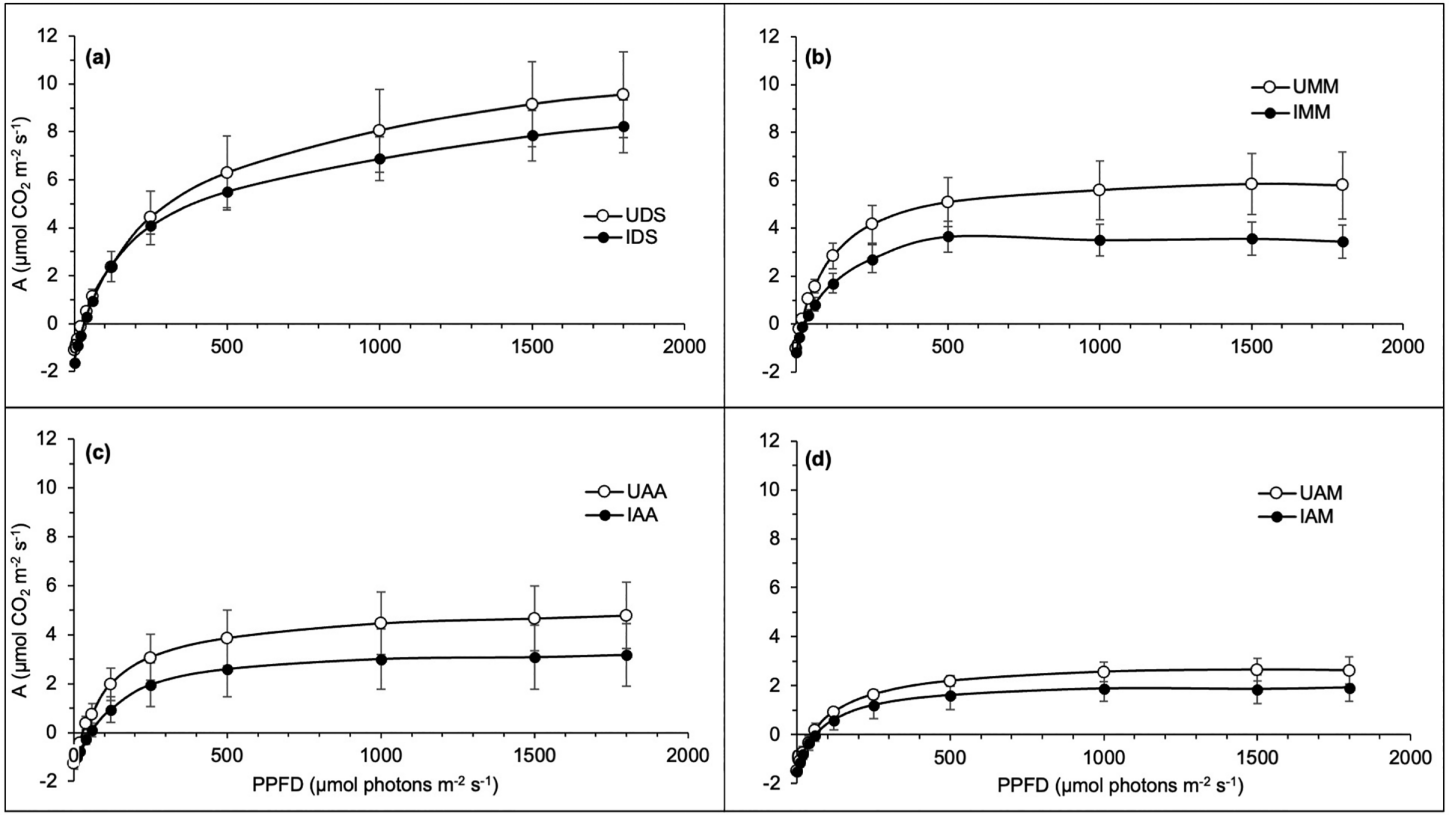
Photosynthetic light response (*A*–*Q*) curves of uninfected (open circle) and infected hosts (black circle) infected by *Cassytha filiformis*: (a) *Dillenia suffruticosa* (DS), (b) *Melastoma malabathricum* (MM), (c) *Acacia auriculiformis* (AA), (d) *Acacia mangium* (AM). The data are expressed as mean values ± standard error, SE with *n* = 6, except for UAM (*n* = 3).

All *Cassytha*‐infected hosts indicated lower photosynthetic performance, *A*, than uninfected hosts in the light response curves (*A*–*Q*) shown in Figure 3. The photosynthetic rate for *D. suffruticosa* continuously increased with elevated light intensity (PPFD), suggesting the light saturation point has not been reached yet (Figure 3a). In contrast, the *A*–*Q* for both infected and uninfected *M. malabathricum*, *A. auriculiformis*, and *A. mangium* reached a plateau at about 1000 μmol photons m^−2^ s^−1^ (Figure 3b–d), compared to those of *D. suffruticosa* (Figure 3a), thus attaining light saturation point at low light intensity. Light saturation point refers to the point where increasing PPFD ceases to result in an increase in photosynthesis. The *A*–*Q* curves of both infected and uninfected exotic *A. mangium* were the lowest (Figure 3d) compared to the photosynthetic performances of the other three hosts at both parasitized and unparasitized scenarios. The light compensation point is the minimum light intensity at which the organism shows a gain of carbon fixation where the CO_2_ assimilated by photosynthesis corresponds with the CO_2_ produced by the processes of light respiration and photorespiration, and thus, net photosynthesis rate is zero. A similar pattern was observed for all host species where the light compensation points of infected hosts occurred at higher PPFD than those uninfected.

**FIGURE 4:**
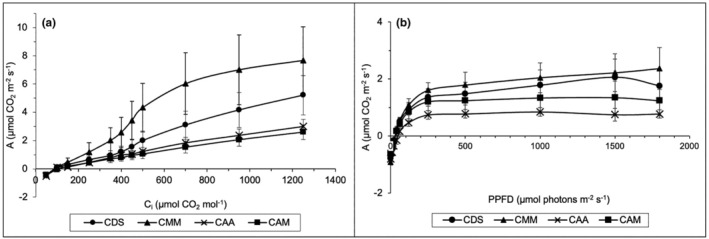
Photosynthetic (a) CO_2_ response (*A*–*C*_i_) and (b) light response (*A*–*Q*) curves of *Cassytha filiformis* parasitizing different native hosts: CDS, CMM, CAA, and CAM. The data are expressed as mean values ± standard error, SE with *n* = 6.

Figure 4a,b depicts the *A*–*C*_i_ and *A*–*Q* curves of *Cassytha* on the studied hosts, that is, native *D. suffruticosa*, CDS; *M. malabathricum*, CMM; the introduced *A. auriculiformis*, CAA; and *A. mangium*, CAM. In the *A*–*C*_i_ curves, the photosynthetic capacities, *A*, of all Cassytha samples increased as the *C*_i_ values elevated (Figure 4a). Interestingly, *Cassytha*‐infecting native hosts (CDS and CMM) exhibited higher *A* than those of the exotic hosts (CAA and CAM). Similar patterns were observed in the *A*–*Q* curves, with CMM having the highest *A*, followed by CDS, CAM, and CAA (Figure 4b). However, instead of reaching a saturation point, all parasite samples exhibited irregular responses when the PPFD was beyond 1000 μmol photons m^−2^ s^−1^. While photosynthetic activity in CMM continued to increase with rising PPFD, *A* in CDS and CAM lowered after 1500 μmol photons m^−2^ s^−1^. CAA showed a decrease in *A* after 1000 μmol photons m^−2^ s^−1^. Albeit the relatively low values of net photosynthetic rate, the light compensation points for the sampled *Cassytha* took place between 30 and 50 μmol photons m^−2^ s^−1^.

### 3.2 Gas exchange measurements and *F*_v_/*F*_m_ ratio of host and parasitic plants

**FIGURE 5:**
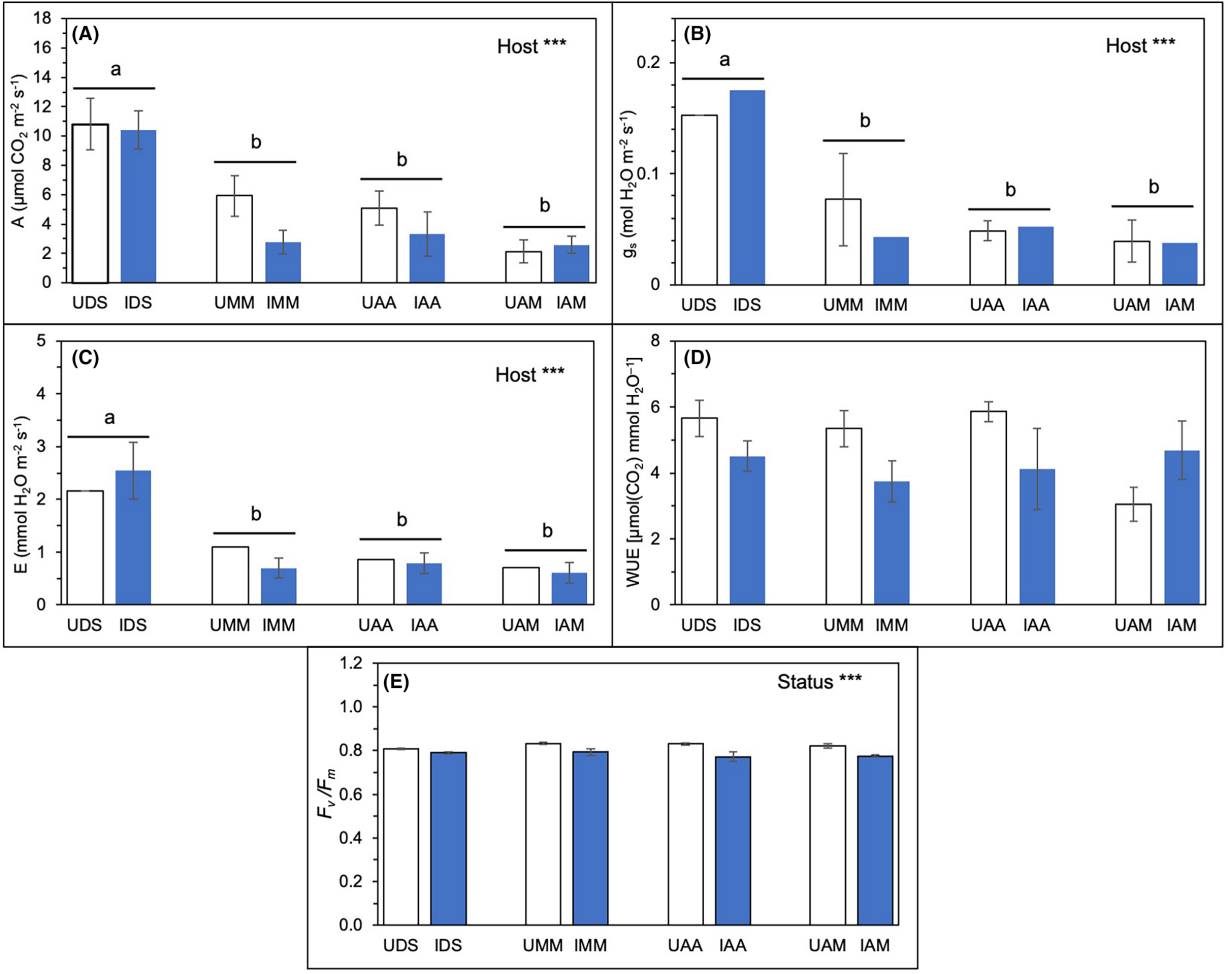
The effects of host species (DS, MM, AA, and AM) represented as “Host” and infection status (infected or I; blue bars and uninfected or U; open bars) represented as “Status” on (A) *A*, μmol CO_2_ m^−2^ s^−1^; (B) *g*_s_; (C) *E*, mmol H_2_O m^−2^ s^−1^, (D) WUE, μmol CO_2_ mmol^−1^ H_2_O, (E) maximum quantum efficiency yield or *F*_v_/*F*_m_, at 5% significance level, which was indicated by ***p < .001 and those not indicated are not significant. Different letters indicate significant differences between host species. The data were expressed as means ± standard error, SE with *n* = 6, except for UMM (*n* = 5) and UAM (*n* = 4).

The variations in instantaneous gas exchange parameters (*A*, *g*_s_, *E*, and WUE) and chlorophyll fluorescence (*F*_v_ */F*_m_) based on “Host” and “Status” are shown in Figure 5 and Table 2. *D. suffruticosa* had significantly higher values in *A*, *g*_s_, and *E* than the other three studied hosts, regardless of their infection status (Figure 5A–C; Table 2). In terms of *F*_v_ */F*_m_ ratio, there was a significant difference based on the infection status in which the chlorophyll fluorescence in uninfected hosts (UDS, UMM, UAA, and UAM) were significantly higher for all hosts than those parasitized by *Cassytha* (IDS, IMM, IAA, and IAM; Figure 5E; Table 2). However, WUE was not significantly influenced by hosts and status (Figure 5D; Table 2).

**TABLE 2:** *p*‐values of two‐way ANOVA on the effects of host species (DS, MM, AA, and AM) represented as “Host” and infection status (infected vs. uninfected) represented as “Status” on gaseous exchange parameters (*A*, *g*_s_, *E*, WUE), chlorophyll a fluorescence *F*_v_ */F*_m_, and total leaf mineral concentrations (N, P, Mg, Ca, K).

Factor	*A*	*g*_s_	*E*	WUE	*F*_v_/*F*_m_	N	P	Mg	Ca	K
Host	**<.001**	**<.001**	**<.001**	.527	.514	**<.001**	**<.001**	.156	**<.001**	**<.001**
Status	.083	.738	.682	.096	**<.001**	.062	.274	**.026**	.876	**.034**

*Note*: Bold values denote statistical significance at the *p* < .05 level.

**FIGURE 6:**
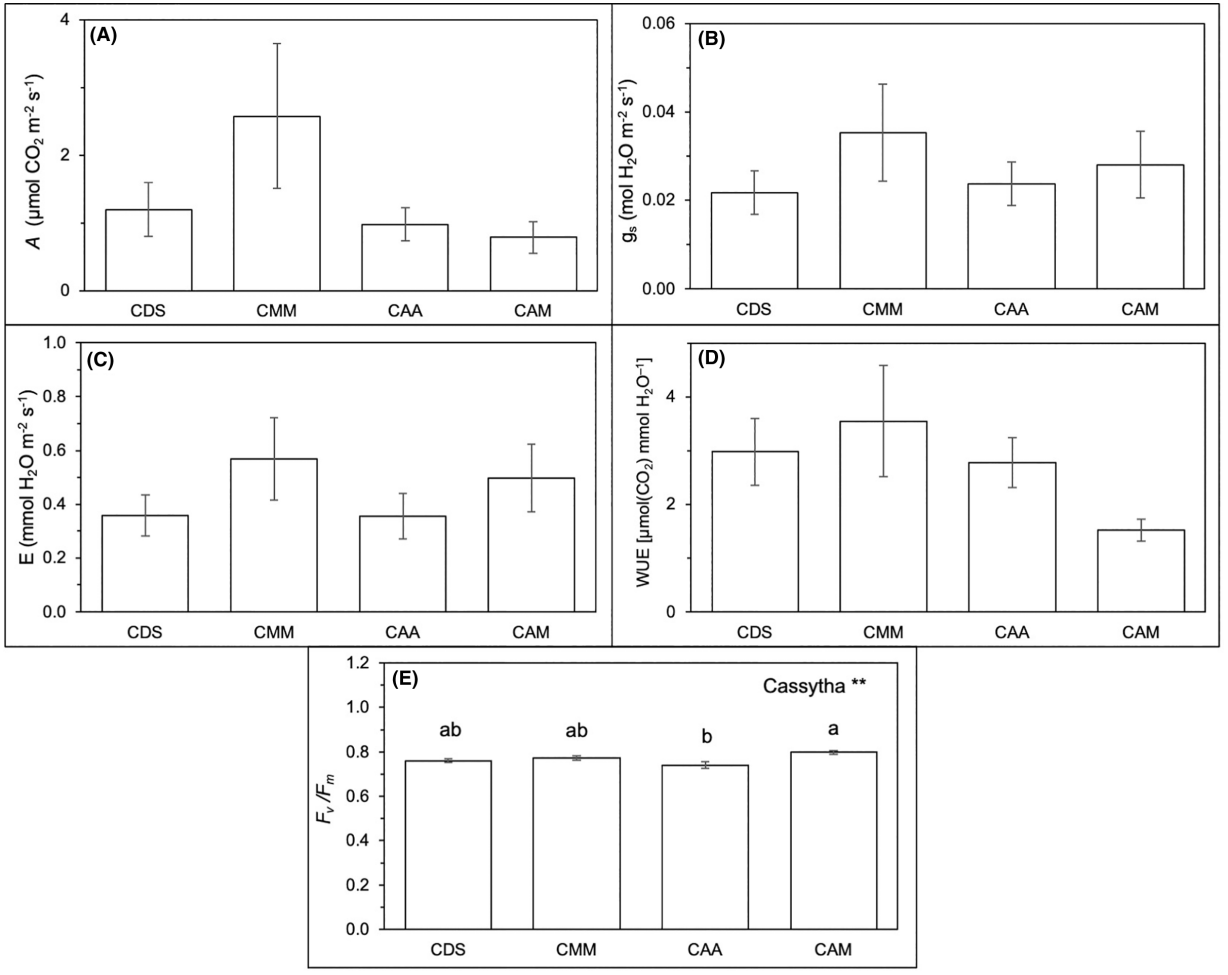
The effects of *Cassytha filiformis* parasitizing the respective studied hosts (CDS, CMM, CAA, and CAM) represented as “Cassytha” and the origin of the infected host (i.e., native for CDS and CMM, and introduced for CAA and CAM) represented as “Origin” on (A) *A*, μmol CO_2_ m^−2^ s^−1^; (B) *g*_s_; (C) *E*, mmol H_2_O m^−2^ s^−1^; (D) WUE, μmol CO_2_ mmol^−1^ H_2_O; (E) maximum quantum efficiency yield or *F*_v_ */F*_m_, at 5% significance level, which was indicated by **p < .01 and those not indicated are not significant. Different letters indicate significant differences between *Cassytha* on various host species. The data were expressed as means ± standard error, SE with *n* = 6.

Similar parameters were evaluated for *Cassytha* on various hosts (CDS, CMM, CAA, and CAM) as shown in Figure 6 and Table 3. None of the instantaneous gas exchange parameters (*A*, g_s_, *E*, and WUE) were affected by the origin of infected hosts (“Origin”) and *C. filiformis* parasitizing the respective studied hosts (“*Cassytha*”) (Figure 6A–D; Table 3). Significant difference was only found in *F*_v_ */F*_m_ ratio among the *Cassytha* samples where CAA showed significantly lowered *F*_v_ */F*_m_ ratio at 0.741 than CAM (0.798) but similar to those infecting the native hosts, CDS (0.761) and CMM (0.772) (Figure 6D; Table 3).

**TABLE 3:** *p*‐values of two‐way ANOVA on the effects of *Cassytha filiformis* parasitizing the respective studied hosts (CDS, CMM, CAA, CAM) represented as “*Cassytha*” and the infected host's origin (i.e., native for CDS and CMM, and introduced for CAA and CAM) represented as “Origin” on *A*, *g*_s_, *E*, WUE, and chlorophyll a fluorescence, *F*v*/F*m and total leaf mineral concentrations (N, P, Mg, Ca, K).

Hosts	*A*	*g*_s_	*E*	WUE	*F*_v_/*F*_m_	N	P	Mg	Ca	K
*Cassytha*	.278	.419	.315	.345	**.004**	.304	**.001**	**.010**	**.029**	**<.001**
Origin	.108	.727	.753	.103	.795	**.032**	**<.001**	**.001**	.900	**<.001**

*Note*: Bold values denote statistical significance at the *p* < .05 level.

### 3.3 Selected nutrient contents in host and parasitic plants

**FIGURE 7:**
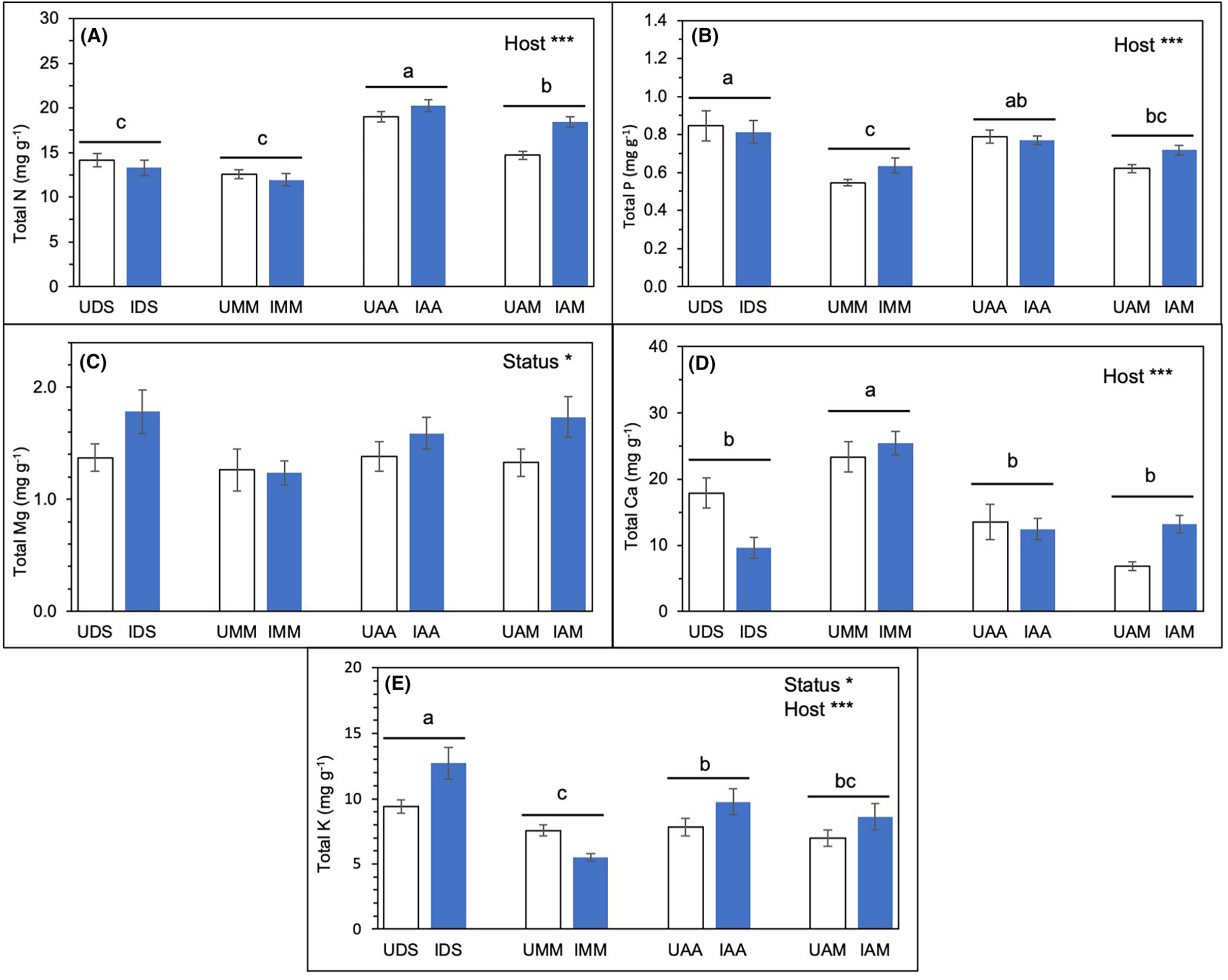
The effects of host species (DS, MM, AA, and AM) represented as “Host” and infection status (infected or I; blue bars and uninfected or U; open bars) represented as “Status” on (A) N, (B) P, (C) Mg, (D) Ca, (E) K, at 5% significance level, which was indicated by *p < .05; ***p < .001 and those not indicated are not significant. Different letters indicate significant differences between host species. The data were expressed as means ± standard error, SE with *n* = 6.

The effects of hosts were significant on total N (Figure 7A; Table 2). *A. auriculiformis* (IAA and UAA) was significantly higher in N (mean = 19.6 mg g^−1^) than *A. mangium* (IAM and UAM) (mean = 16.6 mg g^−1^) and both native hosts, irrespective of their infection status. Total N in AM was also significantly greater than that in the native hosts. There were no significant differences in N between native hosts (mean in DS and MM = 13.7 and 12.3 mg g^−1^, respectively).

Total P in DS was significantly higher than that in the native MM, but similar to AA and AM, regardless of infection status (Figure 7B; Table 2). However, status had an important effect on total Mg as infected hosts (mean = 1.59 mg g^−1^) were significantly high than those uninfected hosts (mean = 1.34 mg g^−1^) (Figure 7C; Table 2). The effects of hosts and status were significant on total K (Figure 7E; Table 2). In terms of hosts, the total K in DS was more significant than those of other host species. For host status, higher K content in infected hosts (mean = 9.15 mg g^−1^) was significant than those uninfected hosts (mean = 7.94 mg g^−1^), albeit varied data were observed in MM, which was significantly lower than the rest and greater total K in UMM than IMM. The effects of hosts showed significant differences in total Ca (Figure 7D; Table 2). Total Ca in MM was significantly higher than that in other studied hosts. Additionally, total Ca in infected MM and AM were higher than the uninfected ones, while it was the opposite in the other two hosts.

**FIGURE 8:**
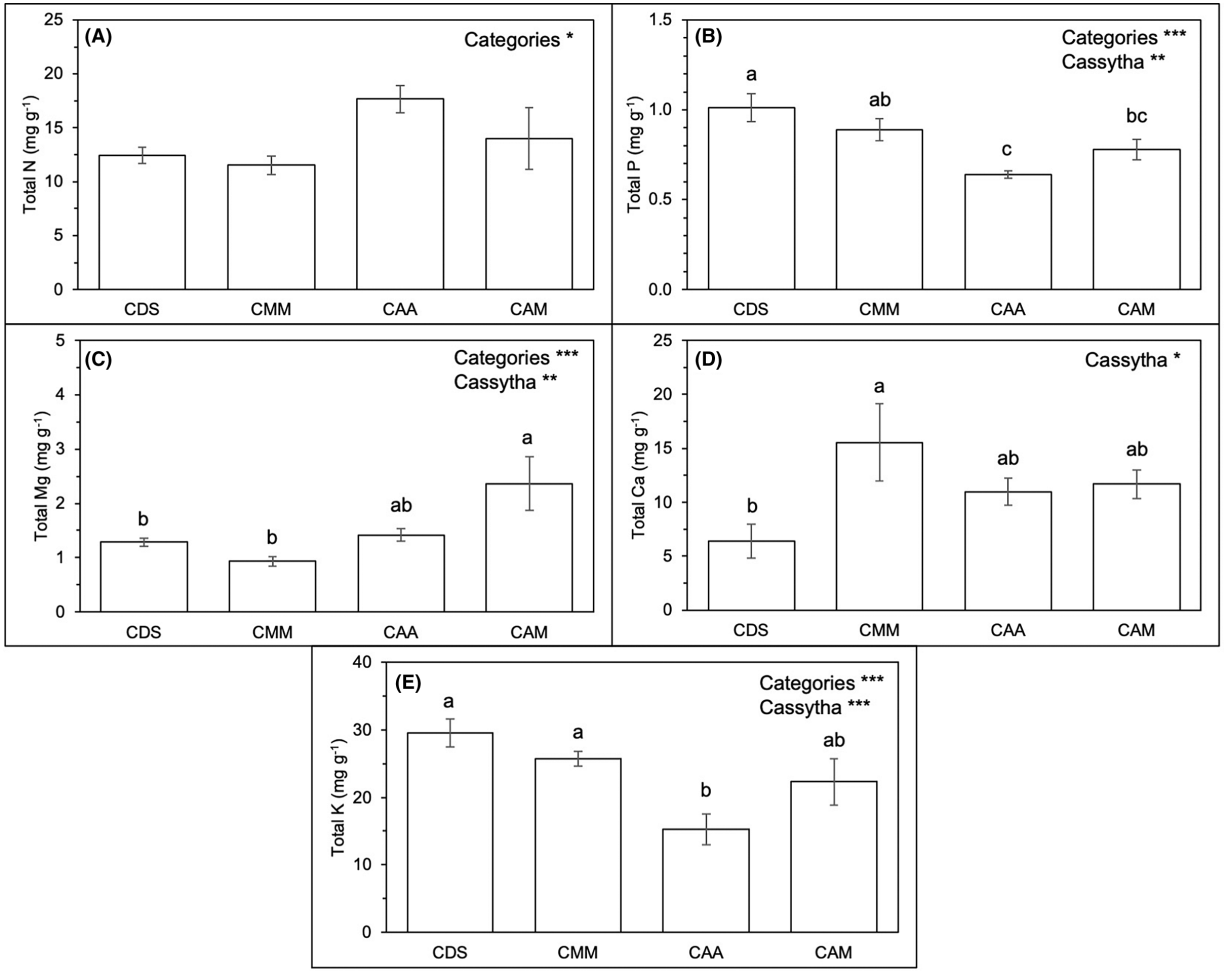
The effects of *Cassytha filiformis* parasitizing the respective studied hosts (CDS, CMM, CAA, and CAM) represented as “Cassytha” and the origin of the infected host (i.e., native for CDS and CMM, and introduced for CAA and CAM) represented as “Origin” on (A) N, (B) P, (C) Mg, (D) Ca, (E) K, at 5% significance level, which was indicated by *p < .05; **p < .01; ***p < .001 and those not indicated are not significant. Different letters indicate significant differences between *Cassytha* on various host species. The data were expressed as means ± standard error, SE with *n* = 6, except for CAM (*n* = 4) in total Mg and Ca, CMM (*n* = 5), and CAM (*n* = 3) in total K.

Similar nutrient profiles were assessed for *Cassytha* on various hosts (CDS, CMM, CAA, and CAM) as shown in Figure 8 and Table 3. The effects of origin (“Origin”; native vs. introduced) of the infected hosts (i.e., native for CDS and CMM, and introduced for CAA and CAM) were significant in terms of N, P, Mg, and K contents (Figure 8A–C,E; Table 3). *Cassytha*‐infecting introduced species (CAA and CAM; mean N = 15.8 mg g^−1^ and mean Mg = 1.89 mg g^−1^) had significantly higher N and Mg than in *Cassytha*‐infecting native species (CDS and CMM; mean N = 12.0 mg g^−1^ and mean Mg = 1.11 mg g^−1^) (Figure 8A,C; Table 3). *Cassytha* that parasitized native host species (CDS and CMM) had significantly greater content in P and K than the introduced hosts (CAA and CMM) (Figure 8B,E; Table 3). Among individual *Cassytha* samples, total P in CDS was similar to CMM but significantly greater than in CAM and CAA (Figure 8B). CAM had significantly higher Mg than CDS and CMM (Figure 8C). It is interesting to note that Ca in CMM was significantly higher than that in CDS, but their Mg contents did not show significant differences compared to CAA and CAM (Figure 8C,D). The high content in total K in CDS and CMM was significantly different from CAA (Figure 8E).

### 3.4 Bioactive contents in host and parasitic plants

Total phenolics and tannins of hosts were not significantly affected by the infection status (infected and uninfected) of hosts, but both bioactive contents were significantly affected by host species (DS, MM, AA, and AM) (Table 4a). In terms of host effect, both AA and AM had significantly higher total phenols than those in DS and MM. The tannin content in AM was significantly higher than both native host species (MM and DS) but was similar to AA. In addition, phenols and tannins in MM were significantly higher than that in DS. For *Cassytha*‐infecting host species, no significant differences were found between *Cassytha* and the host's origin (Table 4b). Phenols and tannin in CDS were not detected by the instrument used.

**TABLE 4:** The effects of (a) host species (DS, MM, AA, and AM) represented as “Host” and infection status (infected and uninfected) of hosts represented as “Status” and (b) *Cassytha filiformis* parasitizing the respective studied hosts (CDS, CMM, CAA, CAM) represented as “*Cassytha*” and the origin of the infected host (i.e., native for CDS and CMM, and introduced for CAA and CAM) represented as “Origin” on total phenolics and tannin in mg g^−1^ at 5% significance level.

(a) Status	Host	Phenols (mg g^−1^)	Tannins (mg g^−1^)
Uninfected	*D. suffruticosa*	9.7 ± 1.19	2.97 ± 0.35
Infected	*D. suffruticosa*	10.2 ± 1.58	3.01 ± 0.34
	Mean	10.0 ± 1.39^c^	2.99 ± 0.34^c^
Uninfected	*M. malabathricum*	11.5 ± 0.77	3.92 ± 0.28
Infected	*M. malabathricum*	13.1 ± 0.07	4.28 ± 0.16
	Mean	12.3 ± 0.42^b^	4.10 ± 0.22^b^
Uninfected	*A. auriculiformis*	15.4 ± 0.24	4.51 ± 0.36
Infected	*A. auriculiformis*	15.1 ± 0.23	4.16 ± 0.25
	Mean	15.2 ± 024^a^	4.34 ± 0.30^ab^
Uninfected	*A. mangium*	15.1 ± 0.38	5.25 ± 0.28
Infected	*A. mangium*	16.0 ± 0.08	4.83 ± 0.17
	Mean	15.6 ± 0.23^a^	5.04 ± 0.23^a^
*p* value	Status	.273	.595
	Host	<.001	<.001

(b) Origin	*Cassytha* (infected host)	Phenols (mg g^−1^)	Tannins (mg g^−1^)
Native	*D. suffruticosa*	Not detected	Not detected
Native	*M. malabathricum*	2.51 ± 0.68^a^	0.57 ± 0.19^a^
Introduced	*A. auriculiformis*	5.22 ± 0.88^a^	1.80 ± 0.43^a^
Introduced	*A. mangium*	3.519 ± 1.06^a^	1.22 ± 0.35^a^
*p* value	Origin	.137	.066
	*Cassytha*	.197	.295

*Note*: Since the effects of infection status (infected and uninfected) of hosts in (a) and origin of the infected host (native and introduced) in (b) were nonsignificant, pooled mean values for each host species were used in (a) and (b). Different letters indicate significant differences between mean values of host species. The data were expressed as means ± standard error, SE with *n* = 6, except for infected MM, *n* = 5; CMM, *n* = 4; and CAM, *n* = 5 in total phenolics and CMM, *n* = 4; and CAM *n* = 4 in tannins.

## 4 DISCUSSION

Gaseous exchange measurements are good indicators of plant stress because they are directly related to net photosynthesis (Le et al., 2015; Long & Bernacchi, 2003). Stem hemiparasites are known to suppress host photosynthetic activities (Cirocco et al., 2016b; Shen et al., 2010), and this study observed similar effects in the light (*A*–*Q*) and CO_2_ (*A*–*C*_i_) response curves of native *D. suffruticosa* and *M. malabathricum*, as well as the alien invasive *A. auriculiformis* and *A. mangium*. However, the trend in *A*–*C*_i_ response curves of *A. mangium* slightly deviated from the others. The CO_2_ (*A*–*C*_i_) response curves begin with low CO_2_ availability (i.e., from 50 μmol mol^−1^), limiting the plants' photosynthesis but increasing the RuBisCO concentration. Minor changes in N values do not impact the photosynthesis of the hosts, directly attributable to the fact that N is a key component of RuBisCO. As CO_2_ concentration gradually increased, the photosynthesis rate also elevated because CO_2_ acts as a substrate for the enzyme, thus elevating the enzyme's carboxylation reaction (Sharkey et al., 2007).

At lower intercellular CO_2_ levels (<500 μmol mol^−1^), infected *A. mangium* performed better photosynthetically than uninfected hosts, suggesting the highly adaptable nature of *A. mangium* (Le et al., 2018) may have enabled it to perform better while under the stress of parasitism and thus prevent energy loss via photorespiration at low CO_2_ (Körner, 2006; Thompson et al., 2017). Under heat stress, parasitized *A. mangium* showed higher survival rates than native species (Ibrahim et al., 2022; Le et al., 2018). Comparably, having a high assimilation rate with limited CO_2_ may be an advantageous strategy of *A. mangium* against native pioneers such as *D. suffruticosa* and *M. malabathricum*. At the same time, identifying the exact mechanisms by which the parasite affects host photosynthesis can be complex (Shen et al., 2010).

In this current study, instantaneous photosynthetic parameters were unaffected by the infection status (infected vs. uninfected), species, and host origin (native vs. exotic) of hosts and *Cassytha*–host species. Although the differences were not significant, instantaneous photosynthetic parameters such as *A*, *g*_s_, and *E* were mainly lower in infected hosts than uninfected ones. Additionally, these parameters were higher in native species than in introduced species when simulated under natural environmental conditions. These findings are similar to studies reported by Shen et al. (2010), whereby *A*, *g*_s_, *E*, and pre‐dawn quantum yields were significantly lower in hosts infected by *C. pubescens* than in uninfected hosts.

Chl a fluorescence analysis is a valuable tool for gauging the performance of the photosynthetic apparatus in response to environmental changes (Strasser et al., 2000; von Caemmerer & Farquhar, 1981), such as parasitic infection in this case. The current study has shown a significant difference in the *F*_v_/*F*_m_ ratio between infected and uninfected hosts, with infected hosts having a significantly lower *F*_v_/*F*_m_ ratio (0.773–0.794). Although no significant difference was detected between native and introduced hosts in our study, the *F*_v_/*F*_m_ ratio of *C. filiformis*‐infected *A. auriculiformis*, an introduced species, was significantly lower (0.741) than *Cassytha*‐infecting native species and a more common *Acacia* species in Brunei, *A. mangium* (0.761–0.798). Our findings coincided with Cirocco et al. (2016b), who found a reduction in *F*_v_/*F*_m_ in the introduced species *U. europaeus* infected by *C. pubescens*, unaffected by high water availability. Our study did not include water availability as a factor; however, Cirocco et al. (2016b) also acknowledged the potential influence of other environmental factors, such as light, at the study site.

In general, it has been observed that *Cassytha* infestation has had significant negative impacts on the photosynthetic parameters compared to other host plants. This may be attributed to the light‐demanding behavior of native heath species (Davies & Semui, 2006; Slik, 2009), as reported by Ibrahim et al. (2022). It may be related to the variability in leaf size, with native plants often producing large, thin leaves and possessing high specific leaf area (SLA) (Dingkuhn et al., 2001). Their SLA tends to increase higher than invasive species when light is limiting (Zhang, Chen et al., 2022), to improve their photosynthetic performances. In the case of *D. suffruticosa*, heavy infestation by *C. filiformis* may have created shady conditions, negatively affecting its photosynthesis. However, this is beyond the scope of this research and there have not been any reports on the effect of parasitism on hosts with various leaf traits, other than the investigation of host size (Cirocco et al., 2020). On the other hand, the native *M. malabathricum* has shown inconsistent results when compared to *D. suffruticosa*, while exhibiting responses more similar to the invasive species. This could be attributed to the “ontogenetic plasticity” of *M. malabathricum* as a pioneer species (Faravani & Bakar, 2007).

The findings from this study indicate that *C. filiformis* parasitism affects various ecophysiological parameters of host plants, but the extent of these effects varies between native and introduced species. Notably, *A. mangium* showed a unique adaptability, maintaining higher photosynthetic performance under parasitic stress, which highlights its resilience compared to other hosts. In contrast, native species generally exhibited greater susceptibility to parasitism, reflected in lower photosynthetic parameters. These results suggest that *C. filiformis* has evolved to co‐exist with native hosts without causing significant harm, implying a possible ecological balance in natural settings.

Future research should focus on controlled experimental setups to isolate the specific impacts of parasitism on photosynthesis, and an integrated approach that includes water relations, biomass, and growth data to provide a comprehensive understanding of the host–parasite dynamics. Despite the limitations of field studies, this research provides valuable insights into the physiological and ecological effects of *Cassytha* parasitism, emphasizing the need for long‐term in situ studies to fully capture the complex interactions in natural habitats.

## CONFLICT OF INTEREST STATEMENT

The authors declare that there is no conflict of interest.

## Supporting information


Tables S1–S4.

## Data Availability

The data that support the findings of this study are available in the supplementary material of this article.

## References

[pei370000-bib-0001] Ainsworth, E. A. , & Gillespie, K. M. (2007). Estimation of total phenolic content and other oxidation substrates in plant tissues using Folin–Ciocalteu reagent. Nature Protocols, 2(4), 875–877. 10.1038/nprot.2007.102 17446889

[pei370000-bib-0002] Allen, S. E. , Grimshaw, H. M. , Parkinson, J. A. , & Quarmby, C. (1989). Chemical analysis of ecological materials. Blackwell Scientific Publications.

[pei370000-bib-0003] Armania, N. , Yazan, L. S. , Musa, S. N. , Ismail, I. S. , Foo, J. B. , Chan, K. W. , Noreen, H. , Hisyam, A. H. , Zulfahmi, S. , & Ismail, M. (2013). *Dillenia suffruticosa* exhibited antioxidant and cytotoxic activity through induction of apoptosis and G2/M cell cycle arrest. Journal of Ethnopharmacology, 146(2), 525–535. 10.1016/j.jep.2013.01.017 23353897

[pei370000-bib-0004] Awang, K. , Conran, J. G. , & Waycott, M. (2018). Cuticular and ultrastructure characters on *Cassytha L*. (Lauraceae) Stem. https://www.researchgate.net/publication/325870821_Cuticular_and_Ultrastructure_Characters_on_Cassytha_L_Lauraceae_Stem/citations

[pei370000-bib-0007] Cirocco, R. M. , Facelli, J. M. , & Watling, J. R. (2016a). High water availability increases the negative impact of a native hemiparasite on its non‐native host. Journal of Experimental Botany, 67(5), 1567–1575. 10.1093/jxb/erv548 26703920 PMC4762389

[pei370000-bib-0008] Cirocco, R. M. , Facelli, J. M. , & Watling, J. R. (2016b). Does light influence the relationship between a native stem hemiparasite and a native or introduced host? Annals of Botany, 117(3), 521–531. 10.1093/aob/mcv193 26832961 PMC4765548

[pei370000-bib-0009] Cirocco, R. M. , Facelli, J. M. , & Watling, J. R. (2017). Does nitrogen affect the interaction between a native hemiparasite and its native or introduced leguminous hosts? New Phytologist, 213(2), 812–821. 10.1111/nph.14181 27717020

[pei370000-bib-0010] Cirocco, R. M. , Facelli, J. M. , & Watling, J. R. (2018). A native parasitic plant affects the performance of an introduced host regardless of environmental variation across field sites. Functional Plant Biology, 45(11), 1128. 10.1071/fp17358 32290974

[pei370000-bib-0011] Cirocco, R. M. , Waterman, M. J. , Robinson, S. A. , Facelli, J. M. , & Watling, J. R. (2015). Native hemiparasite and light effects on photoprotection and photodamage in a native host. Functional Plant Biology, 42(12), 1168. 10.1071/fp15132 32480754

[pei370000-bib-1009] Cirocco, R. M. , Facelli, J. M. , & Watling, J. R. (2020). The impact of a native hemiparasite on a major invasive shrub is affected by host size at time of infection. Journal of Experimental Botany, 71(12), 3725–3734. 10.1093/jxb/eraa140 32185377 PMC7307848

[pei370000-bib-0012] Dale, H. , & Press, M. C. (1998). Elevated atmospheric CO_2_ influences the interaction between the parasitic angiosperm *Orobanche minor* and its host *Trifolium repens* . New Phytologist, 140(1), 65–73. 10.1046/j.1469-8137.1998.00247.x

[pei370000-bib-0013] Davies, S. J. , & Semui, H. (2006). Competitive dominance in a secondary successional rainforest community in Borneo. Journal of Tropical Ecology, 22(1), 53–64.

[pei370000-bib-0014] de la Harpe, A. , Grobbelaar, N. , & Visser, J. (1980). The ultrastructure of the chloroplast and the chlorophyll content of various South African parasitic flowering plants. Zeitschrift für Pflanzenphysiologie, 100(1), 85–90. 10.1016/s0044-328x(80)80189-9

[pei370000-bib-0015] de la Harpe, A. , Visser, J. , & Grobbelaar, N. (1979). The chlorophyll concentration and photosynthetic activity of some parasitic flowering plants. Zeitschrift für Pflanzenphysiologie, 93(1), 83–87. 10.1016/s0044-328x(79)80144-0

[pei370000-bib-0016] de la Harpe, A. , Visser, J. , & Grobbelaar, N. (1981). Photosynthetic characteristics of some South African parasitic flowering plants. Zeitschrift für Pflanzenphysiologie, 103(3), 265–275. 10.1016/s0044-328x(81)80159-6

[pei370000-bib-1006] Din, H. , Metali, F. , & Sukri, R. S. (2015). Tree diversity and community composition of the Tutong white sands, Brunei Darussalam: A rare tropical heath forest ecosystem. International Journal of Ecology, 2015, 1–10. 10.1155/2015/807876

[pei370000-bib-0018] Dingkuhn, M. , Tivet, F. , Siband, P. , Asch, F. , Audebert, A. , & Sow, A. (2001). Varietal differences in specific leaf area: A common physiological determinant of tillering ability and early growth vigor? In S. Peng & H. Bill (Eds.), Rice research for food security and poverty alleviation (pp. 95–108). IRRI. ISBN 971‐220‐157‐0, *International rice research conference*, Los Banos, Philippines.

[pei370000-bib-0020] Faravani, M. , & Bakar, B. B. (2007). Effects of light on seed germination, growth pattern of straits rhododendron (*Melastoma malabathricum* L.). Journal of Agricultural and Biological Science, 2(3), 1–5.

[pei370000-bib-0021] Farquhar, G. D. , & Richards, R. (1984). Isotopic composition of plant carbon correlates with water‐use efficiency of wheat genotypes. Functional Plant Biology, 11(6), 539–552. 10.1071/pp9840539

[pei370000-bib-1001] Fisher, J. P. , Phoenix, G. K. , Childs, D. Z. , Press, M. C. , Smith, S. W. , Pilkington, M. G. , & Cameron, D. D. (2013). Parasitic plant litter input: A novel indirect mechanism influencing plant community structure. New Phytologist, 198(1), 222–231. 10.1111/nph.12144 23356534

[pei370000-bib-0022] Ghazoul, J. , & Sheil, D. (2010). Tropical rain forest ecology, diversity, and conservation. Oxford University Press.

[pei370000-bib-0023] Goh, M. P. Y. , Basri, A. M. , Yasin, H. , Taha, H. , & Ahmad, N. (2017). Ethnobotanical review and pharmacological properties of selected medicinal plants in Brunei Darussalam: *Litsea elliptica*, *Dillenia suffruticosa*, *Dillenia excelsa*, *Aidia racemosa*, *Vitex pinnata* and *Senna alata* . Asian Pacific Journal of Tropical Biomedicine, 7(2), 173–180. 10.1016/j.apjtb.2016.11.026

[pei370000-bib-0028] Ibrahim, M. H. , Sukri, R. S. , Tennakoon, K. U. , Le, Q. V. , & Metali, F. (2022). Photosynthetic responses of invasive *Acacia mangium* and co‐existing native heath forest species to elevated temperature and CO_2_ concentrations. Journal of Sustainable Forestry, 40(6), 573–593. 10.1080/10549811.2020.1792317

[pei370000-bib-0029] Ikbal, I. M. , Din, H. H. , H. Tuah, W. , Md Jaafar, S. , Ahmad, N. , & Sukri, R. S. (2023). Review: Diversity, structure, and community composition of Bornean heath forest with a focus on Brunei Darussalam. Biodiversitas Journal of Biological Diversity, 24(5), 2814–2829. 10.13057/biodiv/d240535

[pei370000-bib-0030] Irving, L. J. , Kim, D. , Schwier, N. , Vaughan, J. K. , Ong, G. , & Hama, T. (2019). Host nutrient supply affects the interaction between the hemiparasite *Phtheirospermum japonicum* and its host *Medicago sativa* . Environmental and Experimental Botany, 162, 125–132. 10.1016/j.envexpbot.2019.02.014

[pei370000-bib-0031] Ismail, N. , & Metali, F. (2014). Allelopathic effects of invasive *Acacia mangium* on germination and growth of local paddy varieties. Journal of Agronomy, 13(4), 158–168. 10.3923/ja.2014.158.168

[pei370000-bib-0032] Jaafar, S. M. , Sukri, R. S. , & Proches, S. (2016). An investigation of soil physico‐chemical variables across different lowland forest ecosystems of Brunei Darussalam. Malaysian Journal of Science, 35(2), 151–168. 10.22452/mjs.vol35no2.6

[pei370000-bib-0033] Jambul, R. , Limin, A. , Ali, A. N. , & Slik, F. (2020). Invasive *Acacia mangium* dominance as an indicator for heath forest disturbance. Environmental and Sustainability Indicators, 8, 100059. 10.1016/j.indic.2020.100059

[pei370000-bib-0035] Körner, C. (2006). Plant CO_2_ responses: An issue of definition, time and resource supply. New Phytologist, 172(3), 393–411. 10.1111/j.1469-8137.2006.01886.x 17083672

[pei370000-bib-0036] Le, Q. , Tennakoon, K. , Metali, F. , Lim, L. , & Bolin, J. (2016). Ecophysiological responses of mistletoe *Dendrophthoe curvata* (Loranthaceae) to varying environmental parameters. Journal of Tropical Forest Science, 28(1), 59–67.

[pei370000-bib-0038] Le, Q. V. , Tennakoon, K. U. , Metali, F. , & Sukri, R. S. (2018). Photosynthesis in co‐occurring invasive *Acacia* spp. and native Bornean heath forest trees at the post‐establishment invasion stage. Journal of Sustainable Forestry, 38(3), 230–243. 10.1080/10549811.2018.1530602

[pei370000-bib-1010] Le, Q. V. , Tennakoon, K. U. , Metali, F. , Lim, B. L. , & Bolin, J. F. (2015). Impact of *Cuscuta australis* infection on the photosynthesis of the invasive host, *Mikania micrantha*, under drought condition. Weed Biology and Management, 15(4), 138–146. 10.1111/wbm.12077

[pei370000-bib-0039] Li, J. , Jin, Z. , & Song, W. (2012). Do native parasitic plants cause more damage to exotic invasive hosts than native non‐invasive hosts? An implication for biocontrol. PLoS One, 7(4), e34577. 10.1371/journal.pone.0034577 22493703 PMC3321012

[pei370000-bib-0040] Li, Y. , & Yao, D. (1992). Anatomical and histochemical studies of haustorial development of *Cassytha filiformis* L. Acta Botanica Sinica, 34, 753–757.

[pei370000-bib-0042] Long, S. P. , & Bernacchi, C. J. (2003). Gas exchange measurements, what can they tell us about the underlying limitations to photosynthesis? Procedures and sources of error. Journal of Experimental Botany, 54(392), 2393–2401. 10.1093/jxb/erg262 14512377

[pei370000-bib-0043] Makkar, H. , Francis, G. , & Becker, K. (2007). Bioactivity of phytochemicals in some lesser‐known plants and their effects and potential applications in livestock and aquaculture production systems. Animal, 1(9), 1371–1391. 10.1017/s1751731107000298 22444893

[pei370000-bib-0045] Maxwell, K. , & Johnson, G. N. (2000). Chlorophyll fluorescence—A practical guide. Journal of Experimental Botany, 51(345), 659–668. 10.1093/jexbot/51.345.659 10938857

[pei370000-bib-0046] Metali, F. , Abu Salim, K. , Tennakoon, K. , & Burslem, D. F. R. P. (2015). Controls on foliar nutrient and aluminium concentrations in a tropical tree flora: Phylogeny, soil chemistry and interactions among elements. New Phytologist, 205(1), 280–292. 10.1111/nph.12987 25138655

[pei370000-bib-0048] Muche, M. , Muasya, A. M. , & Tsegay, B. A. (2022). Biology and resource acquisition of mistletoes, and the defense responses of host plants. Ecological Processes, 11(1), 24. 10.1186/s13717-021-00355-9

[pei370000-bib-0049] Musselman, L. J. , & Press, M. C. (1995). Introduction to parasitic plants. In M. C. Press & J. D. Graves (Eds.), Parasitic plants (pp. 1–13). Chapman & Hall.

[pei370000-bib-0051] Newbery, D. M. (1991). Floristic variation within kerangas (heath) forest: Re‐evaluation of data from Sarawak and Brunei. Vegetatio, 96(1), 43–86. 10.1007/bf00031653

[pei370000-bib-0052] Osunkoya, O. , & Damit, N. (2005). Population dynamics of the invasive Acacias in Brunei Darussalam using matrix modelling. Journal of Physical Therapy Science, 16(2), 115–126.

[pei370000-bib-0054] Press, M. C. , & Phoenix, G. K. (2005). Impacts of parasitic plants on natural communities. New Phytologist, 166(3), 737–751. 10.1111/j.1469-8137.2005.01358.x 15869638

[pei370000-bib-0055] Prider, J. , Watling, J. , & Facelli, J. M. (2009). Impacts of a native parasitic plant on an introduced and a native host species: Implications for the control of an invasive weed. Annals of Botany, 103(1), 107–115. 10.1093/aob/mcn214 19001426 PMC2707288

[pei370000-bib-1003] Prider, J. , Facelli, J. M. , & Watling, J. R. (2011). Multispecies interactions among a plant parasite, a pollinator and a seed predator affect the reproductive output of an invasive plant, *Cytisus scoparius* . Austral Ecology, 36(2), 167–175. 10.1111/j.1442-9993.2010.02132.x

[pei370000-bib-0056] R Core Team . (2022). R: A language and environment for statistical computing. R Foundation for Statistical Computing.

[pei370000-bib-0057] Rosli, R. , Tennakoon, K. U. , Yaakub, M. Y. S. M. , Zainal Ariffin, N. A. H. , & Metali, F. (2024). Host selectivity and distribution of *Cassytha filiformis* in the coastal Bornean heath forests (early view). Tropical Life Sciences Research. 10.21315/tlsr2024.35.2.1 PMC11371412 39234477

[pei370000-bib-1004] Rosli, H. R. (2014). Biology and physiology of the hemiparasitic Cassytha filiformis L . [Master's thesis]. Universiti Brunei Darussalam, Brunei Darussalam.

[pei370000-bib-0059] Sellan, G. , Thompson, J. , Majalap, N. , & Brearley, F. Q. (2022). Influence of species functional strategy on leaf stoichiometric responses to fertilizer in a Bornean heath forest. Journal of Ecology, 110(6), 1247–1258. 10.1111/1365-2745.13865

[pei370000-bib-0060] Sharkey, T. D. , Bernacchi, C. J. , Farquhar, G. D. , & Singsaas, E. L. (2007). Fitting photosynthetic carbon dioxide response curves for C3 leaves. Plant, Cell & Environment, 30(9), 1035–1040. 10.1111/j.1365-3040.2007.01710.x 17661745

[pei370000-bib-0062] Shen, H. , Hong, L. , Ye, W. , Cao, H. , & Wang, Z. (2007). The influence of the holoparasitic plant *Cuscuta campestris* on the growth and photosynthesis of its host *Mikania micrantha* . Journal of Experimental Botany, 58(11), 2929–2937. 10.1093/jxb/erm168 17656466

[pei370000-bib-0063] Shen, H. , Prider, J. N. , Facelli, J. M. , & Watling, J. R. (2010). The influence of the hemiparasitic angiosperm *Cassytha pubescens* on photosynthesis of its host *Cytisus scoparius* . Functional Plant Biology, 37(1), 14–21. 10.1071/fp09135

[pei370000-bib-0064] Slik, J. W. F. (2009). Plants of Southeast Asia. SAS Institute Inc. Retrieved April 15, 2022, from http://www.asianplant.net/

[pei370000-bib-0065] Strasser, R. J. , Srivastava, A. , & Tsimilli‐Michael, M. (2000). The fluorescence transient as a tool to characterize and screen photosynthetic samples. In M. Yunus , U. Pathre , & P. Mohanty (Eds.), Probing photosynthesis: Mechanisms, regulation and adaptation (pp. 445–483). Taylor and Francis.

[pei370000-bib-0071] Těšitel, J. (2016). Functional biology of parasitic plants: A review. Plant Ecology and Evolution, 149(1), 5–20. 10.5091/plecevo.2016.1097

[pei370000-bib-0066] Tang, Y. , & Wang, C. K. (2011). A feasible method for measuring photosynthesis in vitro for major tree species in northeastern China. Chinese Journal of Plant Ecology, 35(4), 452–462. 10.3724/sp.j.1258.2011.00452

[pei370000-bib-0067] Teixeira‐Costa, L. , & Davis, C. C. (2021). Life history, diversity, and distribution in parasitic flowering plants. Plant Physiology, 187(1), 32–51. 10.1093/plphys/kiab279 35237798 PMC8418411

[pei370000-bib-0068] Tennakoon, K. U. , Chak, W. H. , & Bolin, J. F. (2011). Nutritional and isotopic relationships of selected Bornean tropical mistletoe–host associations in Brunei Darussalam. Functional Plant Biology, 38(6), 505–513. 10.1071/fp10211 32480904

[pei370000-bib-0069] Tennakoon, K. U. , Chak, W. H. , Lim, L. B. L. , & Bolin, J. F. (2014). Mineral nutrition of the hyperparasitic mistletoe *Viscum articulatum* Burm. f. (Viscaceae) in tropical Brunei Darussalam. Plant Species Biology, 29(1), 101–107. 10.1111/j.1442-1984.2012.00391.x

[pei370000-bib-0070] Tennakoon, K. U. , Rosli, R. , & Le, Q. V. (2016). Biology of aerial parasitic vines in Brunei Darussalam: *Cuscuta* and *Cassytha* (love vines). Scientia Bruneiana, 15, 58–64. 10.46537/scibru.v15i0.24

[pei370000-bib-1008] Thompson, M. , Gamage, D. , Hirotsu, N. , Martin, A. , & Seneweera, S. (2017). Effects of elevated carbon dioxide on photosynthesis and carbon partitioning: A perspective on root sugar sensing and hormonal crosstalk. Frontiers in Physiology, 8, 578. 10.3389/fphys.2017.00578 28848452 PMC5550704

[pei370000-bib-0073] Toth, G. B. , & Pavia, H. (2001). Removal of dissolved brown algal phlorotannins using insoluble polyvinylpolypyrrolidone (PVPP). Journal of Chemical Ecology, 27(9), 1899–1910. 10.1023/a:1010421128190 11545378

[pei370000-bib-0075] Tuah, W. H. , Tennakoon, K. U. , Jaafar, S. M. , & Sukri, R. S. (2020). Post‐fire impacts on tree diversity in coastal heath forests of Brunei Darussalam. Scientia Bruneiana, 19(1), 19–32. 10.46537/scibru.v19i1.109

[pei370000-bib-0076] von Caemmerer, S. , & Farquhar, G. D. (1981). Some relationships between the biochemistry of photosynthesis and the gas exchange of leaves. Planta, 153(4), 376–387. 10.1007/bf00384257 24276943

[pei370000-bib-0077] Watling, J. R. , & Press, M. C. (2000). Infection with the parasitic angiosperm *Striga hermonthica* influences the response of the C_3_ cereal *Oryza sativa* to elevated CO_2_ . Global Change Biology, 6(8), 919–930. 10.1046/j.1365-2486.2000.00366.x

[pei370000-bib-0079] Weber, J. Z. (1981). A taxonomic revision of *Cassytha* (Lauraceae) in Australia. Journal of the Adelaide Botanic Garden, 3(3), 187–262.

[pei370000-bib-1005] Wong, K. M. , Ahmad, J. , Low, Y. , & Kalat, M. A. (2015). Rainforest plants and flowers of Brunei Darussalam (pp. 150–151). Forestry Department, Ministry of Industry and Primary Resources.

[pei370000-bib-0080] Yu, H. , & Ong, B. L. (2002). The effect of phyllode temperature on gas exchange and chlorophyll fluorescence of *Acacia mangium* . Photosynthetica, 40(4), 635–639. 10.1023/a:1024328808629

[pei370000-bib-0081] Zhang, H. , Florentine, S. , & Tennakoon, K. U. (2022). The angiosperm stem hemiparasitic genus *Cassytha* (Lauraceae) and its host interactions: A review. Frontiers in Plant Science, 13, 864110. 10.3389/fpls.2022.864110 35734256 PMC9208266

[pei370000-bib-0082] Zhang, L. , Chen, A. , Li, Y. , Li, D. , Cheng, S. , Cheng, L. , & Liu, Y. (2022). Differences in phenotypic plasticity between invasive and native plants responding to three environmental factors. Life, 12(12), 1970. 10.3390/life12121970 36556335 PMC9781723

[pei370000-bib-0083] Zoletto, B. , & Cicuzza, D. (2022). Heath forest in tropical Southeast Asia, its ecology and conservation risk. In D. A. DellaSala & M. I. Goldstein (Eds.), Imperiled: The encyclopedia of conservation. Elsevier. 10.1016/B978-0-12-821139-7.00235-X

