# Supplementary material for: Ecophysiological responses of native and introduced coastal tree species parasitized by Cassytha filiformis in Brunei
Source: Plant Environ Interact. 2024 Jul 22;5(4):e70000. doi: 10.1002/pei3.70000 (PMC11261464; doi:10.1002/pei3.70000)
Supplement: Supplementary file 1 — Tables S1–S4. [file PEI3-5-e70000-s001.docx]

**Supporting Information Table S1.** Mean CO_2_ assimilation rate (A) (μmol CO_2_ m^-2^ s^-1^), stomatal conductance (g_s_) (mol H_2_O m^-2^ s^-1^) and transpiration rates (E) (mmol H_2_O m^-2^ s^-1^), water-use efficiency (WUE) (μmol CO_2_ mmol H_2_O^-1^) and chlorophyll *a* fluorescence (F_v_/F_m_ ratio) of host species (*Dillenia suffruticosa*, *Melastoma malabathricum, Acacia auriculiformis,* and *Acacia mangium)* under different infection status (uninfected and infected).

| Status | Host | Assimilation rate (A)  (μmol CO_2_ m^-2^ s^-1^) | Stomatal conductance (g_s_)  (mol H_2_O m^-2^ s^-1^) | Transpiration rate (E)  (mmol H_2_O m^-2^ s^-1^) | Water-use efficiency  (WUE)  (μmol CO_2_ mmol H_2_O^–1^) | F_v_/F_m_ ratio |
| --- | --- | --- | --- | --- | --- | --- |
| Uninfected | *D. suffruticosa* | 10.81 ± 1.78 | 0.15 ± 0.04 | 2.16 ± 0.54 | 5.65 ± 0.54 | 0.808 ± 0.005 |
| Infected |  | 10.42 ± 1.29 | 0.18 ± 0.04 | 2.55 ± 0.53 | 4.51 ± 0.47 | 0.790 ± 0.006 |
| Uninfected | *M. malabathricum* | 5.92 ± 1.39 | 0.077 ± 0.01 | 1.10 ± 0.19 | 5.34 ± 0.54 | 0.832 ± 0.005 |
| Infected |  | 2.76 ± 0.80 | 0.043 ± 0.01 | 0.69 ± 0.13 | 3.74 ± 0.63 | 0.794 ± 0.016 |
| Uninfected | *A. auriculiformis* | 5.10 ± 1.17 | 0.049 ± 0.01 | 0.86 ± 0.20 | 5.86 ± 0.29 | 0.831 ± 0.004 |
| Infected |  | 3.31 ± 1.51 | 0.052 ± 0.02 | 0.80 ± 0.28 | 4.13 ± 1.23 | 0.773 ± 0.022 |
| Uninfected | *A. mangium* | 2.12 ± 0.78 | 0.039 ± 0.01 | 0.70 ± 0.20 | 3.05 ± 0.51 | 0.822 ± 0.009 |
| Infected |  | 2.59 ± 0.56 | 0.038 ± 0.01 | 0.61 ± 0.17 | 4.06 ± 0.89 | 0.775 ± 0.04 |

†The data were expressed as means ± standard error, SE with n=6, except for UMM (n=5) and UAM (n=4).

**Supporting Information Table S2.** Mean CO_2_ assimilation rate (A) (μmol CO_2_ m^-2^ s^-1^), stomatal conductance (g_s_) (mol H_2_O m^-2^ s^-1^) and transpiration rates (E) (mmol H_2_O m^-2^ s^-1^), water-use efficiency (WUE) (μmol CO_2_ mmol H_2_O^-1^) and chlorophyll *a* fluorescence (F_v_/F_m_ ratio) of *Cassytha filiformis* infecting on host species of differing native origin categories i.e. native *Dillenia suffruticosa*, and *Melastoma malabathricum,* as well as the introduced *Acacia auriculiformis,* and *Acacia mangium.*

| Status | Cassytha of infected host | Assimilation rate (A)  (μmol CO_2_ m^-2^ s^-1^) | Stomatal conductance (g_s_)  (mol H_2_O m^-2^ s^-1^) | Transpiration rate (E)  (mmol H_2_O m^-2^ s^-1^) | Water-use efficiency  (WUE)  (μmol CO_2_ mmol H_2_O^–1^) | F_v_/F_m_ ratio |
| --- | --- | --- | --- | --- | --- | --- |
| Native | *D. suffruticosa* | 1.197 ± 0.40 | 0.0218 ± 0.005 | 0.358 ± 0.08 | 2.977 ± 0.62 | 0.761 ± 0.009 |
| Native | *M. malabathricum* | 2.580 ± 1.07 | 0.0354 ± 0.011 | 0.568 ± 0.15 | 3.548 ± 1.03 | 0.772 ± 0.010 |
| Introduced | *A. auriculiformis* | 0.980 ± 0.25 | 0.0237 ± 0.005 | 0.356 ± 0.08 | 2.777 ± 0.47 | 0.741 ± 0.014 |
| Introduced | *A. mangium* | 0.786 ± 0.23 | 0.0281 ± 0.008 | 0.497 ± 0.13 | 1.515 ± 0.21 | 0.798 ± 0.008 |

†The data were expressed as means ± standard error, SE with n=6.

**Supporting Information Table S3.** Mean values of total leaf mineral concentrations i.e. nitrogen, N; phosphorus, P; magnesium, Mg; calcium, Ca; potassium, K (mg g^-1^), as well as total phenolics and tannins (mg g^-1^) of host species (*Dillenia suffruticosa*, *Melastoma malabathricum, Acacia auriculiformis,* and *Acacia mangium)* under different infection status (uninfected and infected).

| Status | Host | Total N  (mg g^-1^) | Total ﻿P  (mg g^-1^) | Total Mg  (mg g^-1^) | Total Ca  (mg g^-1^) | Total K  (mg g^-1^) | Phenolic  (mg g^-1^) | Tannin  (mg g^-1^) |
| --- | --- | --- | --- | --- | --- | --- | --- | --- |
| Uninfected | *D. suffruticosa* | 13.30 ± 0.88 | 0.85 ± 0.08 | 1.37 ± 0.12 | 17.9 ± 2.31 | 9.39 ± 0.51 | 10.2 ± 1.58 | 3.01 ± 0.34 |
| Infected |  | 14.12 ± 0.73 | 0.81 ± 0.06 | 1.78 ± 0.19 | 9.6 ± 1.56 | 12.72 ± 1.21 | 9.7 ± 1.19 | 2.97 ± 0.35 |
| Uninfected | *M. malabathricum* | 11.93 ± 0.69 | 0.55 ± 0.02 | 1.26 ± 0.19 | 23.3 ± 2.28 | 7.56 ± 0.44 | 13.1 ± 0.07 | 4.28 ± 0.16 |
| Infected |  | 12.59 ± 0.47 | 0.64 ± 0.04 | 1.24 ± 0.11 | 25.4 ± 1.76 | 5.50 ± 0.30 | 11.5 ± 0.77 | 3.92 ± 0.28 |
| Uninfected | *A. auriculiformis* | 20.29 ± 0.67 | 0.79 ± 0.03 | 1.39 ± 0.13 | 13.5 ± 2.68 | 7.85 ± 0.68 | 15.1 ± 0.23 | 4.16 ± 0.25 |
| Infected |  | 19.02 ± 0.56 | 0.77 ± 0.02 | 1.59 ± 0.14 | 12.5 ± 1.64 | 9.77 ± 0.99 | 15.4 ± 0.24 | 4.51 ± 0.36 |
| Uninfected | *A. mangium* | 18.44 ± 0.61 | 0.62 ± 0.02 | 1.33 ± 0.12 | 6.8 ± 0.68 | 6.95 ± 0.62 | 16.0 ± 0.08 | 4.83 ± 0.17 |
| Infected |  | 14.71 ± 0.44 | 0.72 ± 0.02 | 1.73 ± 0.18 | 13.2 ± 1.35 | 8.62 ± 1.04 | 15.1 ± 0.38 | 5.25 ± 0.28 |

†The data were expressed as means ± standard error, SE with n=6, except for infected MM, n=5.

**Supporting Information Table S4.** Mean values of total leaf mineral concentrations i.e. nitrogen, N; phosphorus, P; magnesium, Mg; calcium, Ca; potassium, K (mg g^-1^), as well as total phenolics and tannins (mg g^-1^) of *Cassytha filiformis* infecting on host species of differing native origin categories i.e. native *Dillenia suffruticosa*, and *Melastoma malabathricum,* as well as the introduced *Acacia auriculiformis,* and *Acacia mangium.*

| Status | *Cassytha* of infected host | Total N  (mg g^-1^) | Total ﻿P  (mg g^-1^) | Total Mg  (mg g^-1^) | Total Ca  (mg g^-1^) | Total K  (mg g^-1^) | Phenolic  (mg g^-1^) | Tannin  (mg g^-1^) |
| --- | --- | --- | --- | --- | --- | --- | --- | --- |
| Native | *D. suffruticosa* | 12.44 ± 0.74 | 1.01 ± 0.08 | 1.29 ± 0.07 | 6.38 ± 1.55 | 29.56 ± 2.10 | Not detected | Not detected |
| Native | *M. malabathricum* | 11.53 ± 0.87 | 0.89 ± 0.06 | 0.93 ± 0.09 | 15.55 ± 3.58 | 25.76 ± 1.06 | 2.51 ± 0.68 | 0.569 ± 0.19 |
| Introduced | *A. auriculiformis* | 17.66 ± 1.29 | 0.64 ± 0.02 | 1.42 ± 0.11 | 10.98 ± 1.27 | 15.25 ± 2.27 | 5.22 ± 0.88 | 1.803 ± 0.43 |
| Introduced | *A. mangium* | 14.02 ± 2.86 | 0.78 ± 0.06 | 2.37 ± 0.49 | 11.69 ± 1.33 | 22.33 ± 3.45 | 3.51 ± 1.06 | 1.216 ± 0.35 |

†The data were expressed as means ± standard error, SE with n=6, except for CAM (n=4) in total Mg and Ca, CMM (n=5), and CAM (n=3) in total K, CMM (n=4) and CAM (n=5) in total phenolics, and CMM (n=4) and CAM (n=4) in tannins.
